# A randomized, double blind, placebo and active comparator controlled pilot study of UP446, a novel dual pathway inhibitor anti-inflammatory agent of botanical origin

**DOI:** 10.1186/1475-2891-11-21

**Published:** 2012-04-05

**Authors:** John S Sampalis, Lidia Alfaro Brownell

**Affiliations:** 1 JSS Medical Research Inc., Montreal, Quebec, Canada; 2 Surgery and Epidemiology McGill University and Universite du Montreal, Montreal, Quebec, Canada; 3 Unigen Inc., 2260 Willamette Drive NE, 98516 Lacey, Washington, USA

**Keywords:** NSAIDs, Anti-inflammatory, COX-2, LOX, UP446

## Abstract

**Background:**

Current use of prescribed or over the counter non-steroidal anti-inflammatory drugs (NSAIDs) for pain and osteoarthritis (OA) have untoward gastrointestinal and cardiovascular related side effects, as a result the need for a safe and effective alternative has become unequivocally crucial.

**Method:**

A randomized, double blind, placebo and active controlled pilot study of a novel dual pathway, COX1/2 and LOX, inhibitor anti-inflammatory agent of botanical origin, UP446 was conducted. Sixty subjects (age 40-75) with symptomatic OA of the hip or knee were assigned to 4 treatment groups (n = 15); Group A0 (Placebo, CMC capsule), Group A1 (UP446 250 mg/day), Group A2 (UP446 500 mg/day) and Group A3 (Celecoxib, 200 mg/day). MOS-SF-36 and Western Ontario and McMaster University Osteoarthritis Index (WOMAC) data were collected at baseline and after 30, 60 and 90 days of treatment as a measure of efficacy. Erythrocyte sedimentation rate, C-reactive protein, plasma thrombin time (PTT), fructosamine, Hematology, clinical chemistry and fecal occult blood were monitored for safety.

**Results:**

Statistically significant decrease in WOMAC pain score were observed for Group A1 at day 90, Group A2 at 30 and 90 days and Group A3 at 60 and 90 days. Statistically significant decrease in WOMAC stiffness score were observed for Group A1 and Group A2 at 30, 60 and 90 days; but not for Group A0 and Group A3. The mean change in WOMAC functional impairment scores were statistically significant for Group A1 and Group A2 respectively at 30 days (p = 0.006 and p = 0.006), at 60 days (p = 0.016 and p = 0.002) and at 90 days (p = 0.018 and p = 0.002), these changes were not significant for Group A0 and Group A3. Based on MOS -SF-36 questionnaires, statistically significant improvements in physical function, endurance and mental health scores were observed for all active treatment groups compared to placebo. No significant changes suggestive of toxicity in routine hematologies, serum chemistries, liver enzymes or PTT were noted in any of the treatment groups.

**Conclusion:**

Based on current findings UP446 is safe and efficacious alternative to established anti-inflammatory medications for alleviating OA symptoms as measured by the WOMAC Index.

## Introduction

Osteoarthritis (OA) is the most common form of joint disorder and the most frequent cause of musculo-skeletal disability worldwide [1,2]. As the population ages, the number of affected people with OA is expected to reach 60 million by the year 2020 [1]. In the United States, alone, there are 40 million people with this disease who cost the economy an estimated $60 billion yearly [1].

On a biochemical level the primary feature of OA is the accelerated metabolism of arachidonic acid (AA) generated from cell membranes by the action of phospholipase. AA is metabolized by two parallel pathways, cyclooxygenase and 5-lipoxygenase to yield a variety of physiologically active compounds, notably prostaglandins and leukotrienes, respectively. Many of these molecules are involved in the inflammatory response [3].

Present day OA medications act by blocking one or both of the cyclooxygenase (COX-1, COX-2) pathways resulting in reduction of inflammatory mediators such as prostaglandins and prostacyclins. Unfortunately, this therapeutic action is also responsible for most of the toxicity of these agents. It is thought that blocking the COX pathway(s) shunts more AA metabolism down the 5-LOX path with a resultant increase in levels of highly chemotactic and inflammatory leukotrienes [4]. LTB4 has been shown to stimulate osteoclastic bone resorption [5] and has been detected at high levels in the walls of NSAID induced gastric ulcers [6]. LTB4 is also associated with increased production of the pro-inflammatory cytokines TNFα and IL-1β [7,8]

The class of anti-inflammatory agents, called dual pathway inhibitors more recently developed, blocks all three of the primary AA metabolic pathways and seem to have a more benign toxicity profile than traditional medications. [9]. Dual inhibitors seem not to cause GI damage; rather, they show protective effects on GI mucosa. Potent anti-inflammatory action associated with fewer side effects is the desired outcome but needs confirmation from clinical studies.

UP446 is a proprietary, standardized blend of extracts from two botanical sources that have been used medicinally in China and India for more than 1000 years. The extracts contain free B-ring flavonoids and flavans standardized to baicalin and catechin. In preclinical studies it has been shown to inhibit COX-1, COX-2 and 5-LOX and to block several animal models of inflammation [10,11]. The findings of this study suggest that a standardized composition of UP446 has an effect on pain, stiffness and physical function as evaluated by the WOMAC questionnaire.

### Purpose

This study was conducted to compare the effect and safety of two dosages of UP446 as compared with Celecoxib and placebo in subjects with osteoarthritis (OA) of the knee or hip.

## Materials and methods

### Study design

This was a randomized, double blind, placebo and active comparator controlled study. The study was conducted according to ICH guidelines and under independent institutional review board oversight. An independent review board approved the protocol and all subjects were required to provide written informed consent prior to enrollment and administration of medication or any study procedures. Study subjects were recruited from the practices of primary care physicians in Montreal, Quebec. Subjects that met the inclusion/exclusion criteria were randomly assigned to 1 of the 4 study groups (Table 1).

**Table 1:** Subject randomization

***Group***	***Name***	***Sample size***	***Compound***	***Dosage***
**A_0_**	Placebo	n = 15	Placebo	N/A (CMC capsule b.i.d)
**A_1_**	Dose 1	n = 15	UP446	250 mg/day (125 mg b.i.d.)
**A_2_**	Dose 2	n = 15	UP446	500 mg/day (250 mg b.i.d.)
**A_3_**	Dose 3	n = 15	Celecoxib	200 mg/day (100 mg b.i.d.)

Acetaminophen was provided as rescue medication. Subjects were permitted to take up to 1000 mg t.i.d.

### Study Population

Subjects were ambulatory men or women, 40-75 years old and had evidence of measurable symptoms of osteoarthritis of the knee and/or hip requiring the use of acetaminophen, including COX -2 inhibitors, anti-inflammatory agents or opioid analgesics as treatment medications. There were no significant differences between the four groups with respect to demographic characteristics (Table 2).

**Table 2:** Subject demographics

	UP446250 mg/day	UP446500 mg/day	Celecoxib200 mg/day	Placebo
**Total (N)**	15	15	15	15
**Mean (SD) Age**	62.8 (10.8)	54.6 (14.8)	57.6 (12.6)	55.3 (14.3)
**Male N (%)**	5 (33)	6 (40)	5 (33)	6 (40)
**Female N (%)**	10 (67)	9 (60)	10 (67)	9 (60)

### Major inclusion criteria

1. Age between 40 and 75 years; both genders admissible.

2. Evidence of measurable symptoms of osteoarthritis of the knee and/or hip requiring the use of acetaminophen, anti-inflammatory agents or opioid analgesics.

3. Patients were asked to stop use of all other "pain-killers" (except acetaminophen) the week prior to initiation of the trial for washout purposes.

4. Intention to fully participate in study including attending physician appointments during trial.

### Major exclusion criteria

1. Patients with a history of cardiovascular or renal disease, peptic ulcer disease with or without gastrointestinal hemorrhage or perforation or uncontrolled diabetes mellitus were excluded.

2. The concomitant use of NSAIDs, including COX-2 inhibitors, H2 blockers or proton pump inhibitors was not allowed

3. Patients who had begun a new physical therapy regime within three months of screening were also excluded as were those with a history of allergy to flavonoids, NSAIDs, aspirin or acetaminophen

Acetaminophen was provided as rescue medication. Subjects were permitted to take up to 1000 mg t.i.d.

### Measurements

Safety was measured by physical examinations, vital signs, monthly hematology and chemistry laboratory studies, thrombin time, fecal occult blood and treatment emergent adverse event reporting. Efficacy was measured with self-administered questionnaires, specifically the MOS-SF36 and Western Ontario and McMaster University Osteoarthritis Index (WOMAC) that were completed at baseline and after 30, 60, and 90 days of treatment. Laboratory measures of efficacy included ESR, CRP, and fructosamine at baseline and after 90 days of treatment.

### Statistical methods

The primary outcome measure for efficacy was the subjective assessment of pain as measured by the Western Ontario and McMaster University Osteoarthritis Index (WOMAC). Additional functional outcome measurements were evaluated with the Short Form SF-36. The Western Ontario and McMaster University Osteoarthritis Index (WOMAC) has been widely accepted as a valid tool for the classical measurement of osteoarthritis symptom quantification [12]. The Health Assessment Questionnaire Short Form SF-36 has been designed for use in clinical practice and research, health policy evaluations and general population surveys. It includes one multi-item scale that assesses eight health concepts: 1) limitations in physical activities because of health problems; 2) limitations in social activities because of physical or emotional problems; 3) limitations in usual role activities because of physical health problems; 4) bodily pain; 5) general mental health (psychological distress and well-being); 6) limitations in usual role activities because of emotional problems; 7) vitality (energy and fatigue); and 8) general health perceptions.

Within group changes in the efficacy measures were assessed using the paired Student's t-test. Between groups differences with respect to the change in the efficacy measure were assessed with analysis of variance (ANOVA). Multiple-linear regression analysis was used to adjust for the effect of potential confounders. Safety was evaluated by the incidence of adverse events and change in laboratory test results. Relative rate estimates were used to compare the groups with respect to the incidence of adverse events. Paired Student's t-test was used to assess the change in the laboratory test parameters.

Raw/non standardized values for the WOMAC scores were based on a five point Likert scale with 5 response options ranging from 'none' to 'extreme'. A response of 'none' is scored as 0, 'mild' as 1, 'moderate' as 2, 'severe' as 3, and 'extreme' as 4. Scores for each section were summed to produce pain, stiffness, and physical function Scores.

Standardization to a scale between 0 and 100 was used for uniformity and to enhance the appreciation of the magnitudes of changes. The mean percent change and p value from baseline are calculated from the normalized scores.

Statistical Analysis was performed by JSS Medical Research Inc.; Montreal Quebec.

## Results

Sixty subjects were enrolled in the study, 22 (36.7%) men and 38 (63.3%) women, mean (SD) age 57.6 (13.2) years. There were no statistically significant differences between the four groups with respect to demographic characteristics and baseline disease activity.

Of the 60 subjects who enrolled, 52 (87%) completed the study (Table 3). One patient from the UP446 250 mg/day group was removed from the study at 40 days due to urgent requirement for hip surgery. Two subjects in the UP446 500 mg/day group withdrew after 60 days for personal reasons. From the celecoxib 200-mg/day group, 2 patients withdrew at 30 days because of positive fecal occult test and 1 patient withdrew at 90 days for personal reasons. From the placebo group, 2 patients withdrew after 60 days, 1 for personal reasons and 1 because of noticeable reduced flexibility.

**Table 3:** Final subject disposition by group and visit

		Group			
		UP446250 mgs/day	UP446500 mgs/day	Celecoxib 200 mgs/day	Placebo
Visit	Baseline	15	15	15	15
	30 Days	15	15	13	15
	60 Days	14	13	13	13
	90 Days	14	13	12	13

### WOMAC

**Figure 1:**
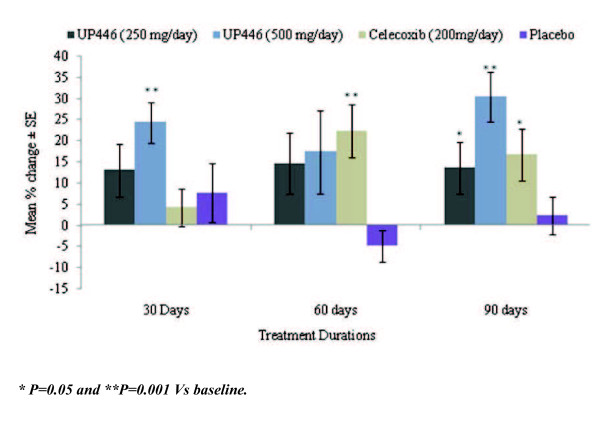
**Change in WOMAC Pain Scores/100 by Visit and Study Group**. Statistical significant changes from baseline were observed during the 90 day study with UP446 500 mgs/day group reaching significance earlier at 30 days.

**Figure 2:**
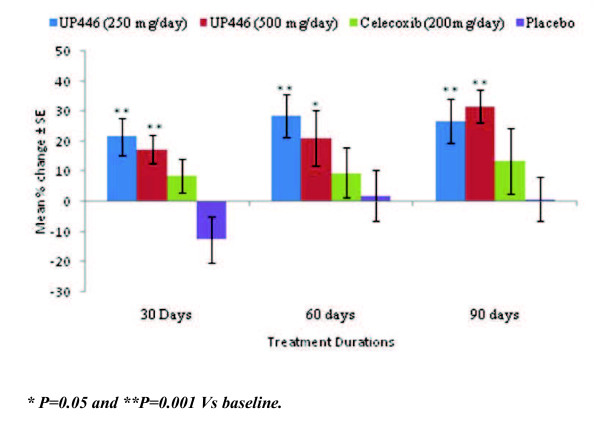
**Change in WOMAC Stiffness Scores/100 by Visit and Study Group**. UP446 250 mgs/day and UP446 500 mgs/day groups had consistently statistical significant changes from baseline during the study.

**Figure 3:**
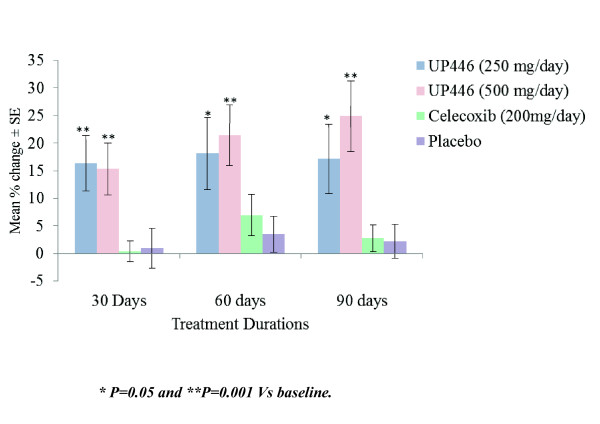
**Change in WOMAC Functional Scores/100 by Visit and Study Group**. Statistical significant change in Functional Scores was only observed in the UP446 treatment groups, UP446 250 mgs/day and UP446 500 mgs/day.

The mean WOMAC scores and mean percent change from baseline at the 30, 60, and 90 day evaluations are summarized in Figures 1, 2 and 3.

#### WOMAC pain scores

Significant decreases in the mean WOMAC pain score were observed for the UP446 250 mg/day group at 90 days (p = 0.045), and for the UP446 500 mg/day group at 30 days (p = 0.001), and 90 days (p = 0.001) but not at 60 days ( p = 0.102). For the Celecoxib group significance was observed at 60 days (p = 0.004) and at 90 days (p = 0.021) but not at 30 days (p = 0.375). The overall within group effect for all visits combined was significant for the UP446 group 500 mgs/day (p = 0.002) and the Celecoxib (p = 0.003) groups only. There were some small changes from baseline for the placebo group but statistical significant was not reached. (Table 4)

**Table 4:** WOMAC pain scores by group and visit

			Group			
			UP446 250 mg/day	UP446 500 mg/day	Celecoxib 200 mg/day	Placebo
Visit	Baseline	Mean	3.17	3.41	3.20	2.97
		Std Deviation	0.80	0.93	0.89	0.60
	30 Days	Mean	2.65	2.44	3.00	2.67
		Std Deviation	0.77	0.92	0.92	0.62
	60 Days	Mean	2.63	2.63	2.20	3.29
		Std Deviation	0.66	0.79	0.66	0.67
	90 Days	Mean	2.67	2.11	2.27	3.00
		Std Deviation	0.65	0.86	0.66	0.58

#### WOMAC stiffness scores

Significant decreases in the mean WOMAC stiffness score were observed for the UP446 250 mg/day group at 30 days (p = 0.004), 60 days (p = 0.002) and 90 days (p = 0.003), and for the UP446 500 mg/day group at 30 days (p = 0.002), 60 days (p = 0.043) and 90 days (p = 0.001). For the celecoxib and placebo groups no significant within group changes were observed (Table 5).

**Table 5:** WOMAC stiffness scores by group and visit

			Group			
			UP446 250 mg/day	UP446 500 mg/day	Celecoxib 200 mg/day	Placebo
Visit	Baseline	Mean	3.53	3.47	2.90	2.87
		Std Deviation	1.08	0.95	0.87	0.85
	30 Days	Mean	2.67	2.77	2.58	3.37
		Std Deviation	0.65	0.84	0.73	0.83
	60 Days	Mean	2.50	2.58	2.50	2.85
		Std Deviation	0.76	0.79	1.19	0.99
	90 Days	Mean	2.57	2.15	2.17	2.96
		Std Deviation	0.83	0.85	1.01	0.72

#### WOMAC functional impairment scores

The mean change in WOMAC functional impairment scores were significant for the UP446 250 mg/day and the UP446 500 mg/day groups respectively at 30 days (p = 0.006 and p = 0.006), at 60 days (p = 0.016 and p = 0.002) and at 90 days (p = 0.018 and p = 0.002). For these two groups the overall within group effect across all visits was significant (p = 0.018) and (p = 0.001) respectively. The changes in functional impairment over time were not significant for the Celecoxib and placebo groups. (Table 6)

**Table 6:** WOMAC functional impairment scores by group and visit

			Group			
			UP446 250 mg/day	UP446 500 mg/day	Celecoxib 200 mg/day	Placebo
Visit	Baseline	Mean	3.34	3.52	2.98	3.11
		Std Deviation	0.91	0.71	0.41	0.33
	30 Days	Mean	2.68	2.90	2.94	3.08
		Std Deviation	0.58	0.69	0.37	0.59
	60 Days	Mean	2.66	2.66	2.65	2.99
		Std Deviation	0.31	0.29	0.36	0.29
	90 Days	Mean	2.70	2.52	2.78	3.04
		Std Deviation	0.68	0.53	0.44	0.51

#### MOS SF-36 questionnaires

The intra-group 30, 60 and 90 SF-36 scores for pain and function in all treatment groups are shown in Tables 7 and 8. Significant improvement in physical function was observed at all follow up visits (p = 0.001) for all groups except the placebo.

**Table 7:** SF-36 pain scale scores by group and visit

		Group				P value(Between Groups)
Visit		UP446 250 mg/day	UP446 500 mg/day	Celecoxib 200 mg/day	Placebo	
Baseline	Mean	8.53	7.53	8.20	7.40	0.272
	Std Deviation	1.85	2.17	1.86	1.24	
30 Days	Mean	7.53	7.67	8.46	7.33	0.325
	Std Deviation	1.41	1.95	.97	2.06	
60 Days	Mean	7.29	7.38	8.08	7.31	0.643
	Std Deviation	1.49	2.06	1.93	1.80	
90 Days	Mean	7.50	6.54	7.50	7.54	0.547
	Std Deviation	2.03	1.98	2.50	1.76	

**Table 8:** SF - 36 Physical function scores by group and visit

			Group				P value(Between Groups)
			UP446 250 mg/day	UP446 500 mg/day	Celecoxib 200 mg/day	Placebo	
Visit	Baseline	Mean	19.53	19.93	21.07	19.40	0.170
		Std Deviation	1.88	2.15	2.19	2.61	
	30 Days	Mean	26.73	27.00	27.00	20.67	0.001
		Std Deviation	1.49	1.07	2.08	1.72	
	60 Days	Mean	27.21	26.77	27.31	21.15	0.001
		Std Deviation	1.31	1.92	1.44	2.08	
	90 Days	Mean	26.36	27.23	25.92	20.23	0.001
		Std Deviation	1.01	1.54	1.00	1.79	

The change in physical role was significant for UP446 250 mgs/day group at 30 days (p = 0.031), 60 days (p = 0.004) and at 90 days (p = 0.041). For the UP446 500 mg/day significant improvement in physical role was observed at 30 days only (p = 0.002). The Celecoxib 200 mgs/day group experienced significant improvement in physical role at 30 days (p = 0.008), at 60 days (p = 0.001) and at 90 days (p =0.004). The placebo group did not have significant changes in physical role. All three of the active treatment groups, but not the placebo group, had significant improvement in the energy/fatigue and mental health scores (p < 0.001). There were no significant changes in the general health scores over time for all study groups.

## Between group analyses

The following sections describe the results of the one -way ANOVA and post-hoc comparisons for between group differences in respect to change in the WOMAC and MOS-SF 36 scale scores.

### WOMAC

In the WOMAC score significant differences were observed at 30, 60 and 90 days in pain while walking on a flat surface. Similar, but less pronounced differences were seen between groups. With respect to pain, UP446 and Celecoxib scored better than placebo at 60 and 90 days. The WOMAC inter-group comparisons for pain, stiffness and function are shown in Table 9. UP446 500 mgs reached statistical significance in the WOMAC pain scale over UP446 250 mgs at 90 days and over placebo at 30, 60 and 90 days. For stiffness UP446 500 mgs was significant at 30 and 90 days over placebo. In the WOMAC function scale UP446 500 mgs showed significance over placebo and Celecoxib at 30, 60 and 90 days.

**Table 9:** WOMAC scales inter-group comparisons

	Pain			Stiffness			Function		
	30 days	60 days	90 days	30 days	60 days	90 days	30 days	60 days	90 days
UP446 500 mgs vs. UP446 250 mgs	NS*	NS	0.038	NS	NS	NS	NS	NS	NS
UP446 250 mgs vs. CELECOXIB 200 mgs	NS	NS	NS	NS	NS	NS	0.010	NS	NS
UP446 500 mgs vs. CELECOXIB 200 mgs	0.020	NS	NS	NS	NS	NS	0.015	0.043	0.039
UP446 250 mgs vs. PLACEBO	NS	NS	NS	0.000	0.027	0.015	0.010	0.043	0.039
UP446 500 mgs vs. PLACEBO	0.044	0.032	0.001	0.001	NS	0.005	0.015	0.016	0.003
CELECOXIB 200 mgs vs. PLACEBO	NS	0.009	NS	0.023	NS	NS	0.015	0.016	0.003

*NS: Not statistically significant

UP446 at 250 mgs was significant over placebo on stiffness at 60 and 90 days and over Celecoxib at 30 days on the function scales. Celecoxib was statistically significant at 60 days over placebo on the pain scale, at 30 days on the stiffness scale and at 30, 60 and 90 days on the Function scale of the WOMAC. (Table 9)

### MOS-SF 36

For the MOS-SF 36, the mean change in the SF-36 function score was significantly different for the four treatment groups at 30, 60 and 90 days ( p = < 0.001). At 30 and 60 days significant differences were observed in physical function score between the placebo group and the active treatment groups (p = < 0.001). Tukey's LSD test showed that at 90 days significant differences existed between UP446 250 mg/day and celecoxib (p = 0.035), UP446 500 mg/day and celecoxib (p = 0.020) and between placebo and the three active treatment groups (p = < 0.001). All three of the active treatment groups, but not the placebo group, had significant improvement in the energy/fatigue and mental health scores (p < 0.001).

Similarly the changes in physical role scores were different between the four groups at 30 days (p = 0.012) 60 days (p = 0.012) and at 90 days (p = 0.009). At 30 days significant differences were observed between the Placebo group and UP446 250 mgs/day (p = 0.015) and celecoxib (p = 0.002). At 60 days the statistically significant comparisons were between UP446 500 mgs/day and celecoxib (p = 0.019), between Placebo and UP446 250 mgs/day (p = 0.024) and between placebo and celecoxib (p = 0.004). Similarly differences were observed at 90 days between UP446 500 mgs/day and celecoxib (p = 0.030) and between placebo and the two UP446 groups (p = 0.020).

The changes in energy/fatigue score were different between the four groups at 30 days (p = 0.001), 60 days (p = 0.011) and at 90 days (p = 0.001). At the 30, 60 and 90 days the significant differences were between the Placebo group and the three active treatment groups (p = 0.001). There were no significant between group differences with respect to pain scores. The change in mental health scores was significantly different between the four groups at 30, 60 and 90 days (p = 0.001). These differences could be due to significantly lower mean changes in the placebo group when compared to the three active treatment groups (p = 0.001).

There were no significant between group differences with respect to the changes in social function, emotional role or general health.

### Laboratory measures of efficacy

C-reactive protein (CRP) data were collected as a marker of inflammation; [13,14]. There was a decrease for the UP446 250 mgs group and the Celecoxib group and a slight increase for the placebo and the UP446 500 mgs group. These changes are not statistically significant for the groups except for the 250 mgs UP446 group change from baseline at 90 days. Most of the changes have little clinical significance due to slight changes that can be attributed to individual and or analytical variability. The second clinical inflammation marker used was the erythrocyte sedimentation rate (ESR). The changes were not statistically or clinically significant for any group all the groups mean values considering the age and gender mix, fall within normal clinical reference range. During the pilot study data was not collected for other markers of inflammation such as cytokines (IL-6, TNF alpha, etc) therefore a conclusion on the specific role of UP446 anti-inflammatory properties cannot be made based on this study [15,16].

The fructosamine levels can give an indication of the average glucose levels over the past 2 to 3 weeks, during the study there were not significant changes between groups but the changes from baseline were significant for all groups. These changes correlated well with the moderate decrease in glucose and body weight changes observed for the groups.

## Safety evaluations

No significant changes were noted in routine hematologies, serum chemistries, liver enzymes or Partial Thrombin time (PTT) in any of the treatment groups.

The PTT was used as a safety marker due to reported inadequate platelet function of drug induced etiology such as aspirin, NSAIDs or liver injury. The PTT values were very consistent for all the groups with no significant or clinical changes from baseline.

The fecal occult blood Test (FOBT) was also used as a safety evaluation of the treatment groups. Nonsteroidal anti-inflammatory drugs (NSAIDs) increase the risk of upper gastrointestinal bleeding. Four NSAIDs commonly used (indomethacin, naproxen, diclofenac, and piroxicam) markedly increased the risk of bleeding: odds ratios range from 4.9 to 19.1 for subjects who used the drug at least once during the week before symptoms began. Aspirin was also associated with a substantially increased risk of bleeding (odds ratio, 7.2); acetaminophen was not [17]. There were 20 subjects with a positive FOBT at the duration of the study. Positive FOBT were distributed among all groups to include the placebo, no frank bleeding was reported. (Table 10)

**Table 10:** Fecal Occult Blood (FOB)

Fecal Occult Blood (FOB)			UP446 250 mgs	UP446 500 mgs	Celecoxib 200 mgs	Placebo
Visit	Baseline	Positive	0	0	0	0
	30 Days	Positive	0	0	2	1
	90 Days	Positive	3	5	5	4
Total per study period			3	5	7	5

In both UP446 groups mean systolic blood pressure decreased from 146 at baseline to 125 at the 30 day visit and remained stable thereafter. This change was not seen in the celecoxib or placebo groups. Diastolic blood pressure and resting pulse did not change during the course of the study.

There were no differences in type or frequency of symptomatic adverse events between the UP446 and placebo groups. Interestingly, all three active treatment groups experienced weight loss, with BMI changes of between 1.6-2.4 kg/m^2^.

It is unclear if this is a metabolic effect of the products or reflects increased activity associated with symptomatic improvement. Other observations noted during adverse effects monitoring for safety are listed below. These events were not classified as treatment related by the Investigator (Table 11).

**Table 11:** Other reported adverse events

Condition	Observations	Treatment
Hypertension	1	UP446 250 mg
Varicose veins	1	UP446 250 mg
Fluid in knee	1	UP446 250 mg
Reduced flexibility	1	Placebo
Psoriasis	2	UP446 250 and 500 mg

## Discussion

This study describes the results of a first pilot clinical trial of UP446 in OA subjects. UP446 is a novel dual pathway inhibitor of botanical origin. The effects of two different doses of this plant extract were evaluated on osteoarthritis and compared to placebo and Celecoxib. This study demonstrated that UP446 at both dose levels and Celecoxib were associated with significant reduction in pain, stiffness and functional incapacity as compared to placebo. Although the treatment groups were small, the results strongly suggest that UP446 at 500 mgs per day was significantly more effective than Celecoxib at 200 mgs for pain reduction and improvement in function as measured by the WOMAC. These findings offer an alternative way of dealing with the discomfort associated with osteoarthritis, there are many NSAIDs available but their major side effects are well known [18]. There are some limitations and interesting findings in this pilot study. For example, the statistical findings in changes from baseline (p value < 0.05) were not consistent at the 30, 60 and 90 days time points for any of the study groups. For the pain the scores, the UP446 at both dose levels has significance at 30 and 90 days but not at 60 days in contrast the celecoxib group at 60 and 90 days but not at 30 days. Review of the study shows that the average change in WOMAC pain score (Figure 1) for UP446 at 250 mgs was remarkably close for the 30, 60 and 90 day measurement; mean -13 at 30 days, -14.64 at 60 days and -13.57 at 90 days. In fact the value at 60 days is if anything suggestive of a better result than the 30 day and 90 day values (Figure 1). We conclude that any "statistical" differences between the values are therefore due to differences in variance (standard deviation), which results in the 60 day value having marginally less statistical significance than the 30 day and 90 day values. Thus while only the 90 day value achieved p < =0.05, in fact the 30 day (p = 0.058) and the 60 day (p = 0.062) values are as meaningful and indicative of efficacy as the 90 day value (p = 0.045). For the UP446 500 mgs/day group statistical significance at p < 0.05 is achieved at 30 days (p = 0.001) and at 90 days (p = 0.001) but not a 60 days (p = 0.102), and for the Celecoxib group significance at p < 0.05 was observed at 60 days (p = 0.004) and at 90 days (p = 0.021) but not a 30 days (p = 0.375).

We also found lack of agreement between the WOMAC and the SF-36 assessments, these are not uncommon and have been previously reported [19], still the WOMAC is the most common and appropriate tool for evaluation of hip and knee osteoarthritis [20].

The fact that there were positive fecal occult blood tests (FOBT) in all the groups to include placebo makes it difficult to use this as a safety marker. Since the total number of positive test results in the placebo group, was equal to or greater than the two UP446 treatment groups (Table 10), the occurrence of positive FOBT test results is not UP446 treatment related. The guaiac test utilizes a colorimetric indicator that produces a detectable color change upon oxidation [21]. The presence of occult blood is based on oxidation of the indicator by the heme moiety of hemoglobin, which possesses peroxidase activity. There are however, a large number of other redox substances and peroxidases or peroxidase-like enzymes that will test positive or otherwise interfere with this assay [22]. Many of these interferants are present in normal dietary foods. While patients may be warned not to eat red meat or certain fruits and vegetables, to avoid use of aspirin or other nonsteroidal anti-inflammatory drugs (NSAIDs), and to avoid ingesting vitamin C, e.g., orange juice, all for the 72 hour period before testing, strict compliance with these instructions is likely poor.

The baseline average systolic blood pressures for the study groups range from a mean of 134 to 159; thus all groups are pre-hypertensive or overtly hypertensive as defined by the American Heart Association. The high baseline systolic blood pressures combined with the reported increased physical activity and the observed weight loss for all three of the treatment groups is likely to account for the beneficial reduction in systolic blood pressure with the result that participants in the treatment groups are closer to desirable blood pressure values as per the American Heart Association Guidelines, less 120 mm Hg Systolic and less than 80 mm Hg Diastolic. Notably maximal reduction in systolic blood pressure was achieved after 30 days dosing with no further reduction observed after dosing for 90 days. Diastolic blood pressure and resting pulse did not change during the course of the study.

All active treatment groups experienced weight loss, with BMI changes of between 1.6-2.4 kg/m^2^. The study participants reported no other adverse events and the clinical laboratory evaluations showed no abnormal results that would be indicative of abnormal nutritional status or metabolism. Per protocol the study participants had no dietary or exercise restrictions. These changes can be attributed to increased physical activity reported by study participants as a result of improved mobility and reduced discomfort associated with treatment regimens.

## Conclusion

In summary the results of the present study provide data that supports the use of UP446 at 250 mg/day and at 500 mg/day for the effective management of the clinical course of osteoarthritis. Significant improvement of osteoarthritis symptoms was noticed after 30, 60 and 90 days of treatment with UP446 at 250 mgs and 500 mgs/day. UP446 at 250 mg/day and 500 mg/day is significantly more effective than Celecoxib 200 mg/day for the reduction of function incapacity caused by osteoarthritis, within 30 days (p = 0.010) of treatment and UP446 500 mgs is more effective than celecoxib for reduction of pain at 30 days (p = 0.020)The proprietary plant extract was well tolerated. Symptomatic adverse events and incident of positive fecal occult blood were comparable to the placebo group. No other serious adverse events were observed.

On the basis of the positive findings in this pilot trial of UP446 we concluded that the mechanism of dual COX/LOX inhibition may confer clinical safety and efficacy benefits comparable to or, in some cases superior to traditional NSAID treatment, offering an effective alternative option in a dietary supplement for managing discomfort associated with OA. Based on the preliminary evidence of this pilot study an additional study in larger patient population is currently underway to further assess the safety and efficacy of UP446.

## Competing interests

LB is an employee of Unigen; JSS received funds to conduct the clinical trial from Unigen.

## Authors' contributions

JS drafted first version of manuscript, participated in the statistical analysis and study conduct. LB revised the manuscript and added additional sections. All authors read and approved final manuscript.

## References

[B1] Felson DT The course of osteoarthritis and factors that affect it Rheum Clin North Am 1993 19 607 615 8210577

[B2] Zhang Y Prevalence of symptomatic hand osteoarthritis and its impact on functional status among the elderly, The Framingham Study Am J Epidemiol 2002 156 1021 1027 10.1093/aje/kwf141 12446258

[B3] Felson Osteoarthritis: New Insights: Part 1: The Disease and Its Risk Factors Ann Intern Med 2000 133 8 635 646 11033593 10.7326/0003-4819-133-8-200010170-00016

[B4] Paredes Y Study of the role of leukotriene B4 in abnormal function of human subchondral osteoarthritis osteoblasts: effects of cyclooxygenase and/or 5-lipoxygenase inhibition Arth Rheum 2002 46 1804 1812 10.1002/art.10357 12124864

[B5] Garcia C Leukotriene B4 stimulates osteoclastic bone resorption both in vitro and in vivo J Bone Miner Res 1996 11 1619 1627 8915769 10.1002/jbmr.5650111105

[B6] Rainsford KD Leukotrienes in the pathogenesis of NSAID-induced gastric and intestinal mucosal damage Agens Actions 1993 39 C24 C26 10.1007/BF01972709 8273575

[B7] Rainsford KD Effects of 5-lipoxygenase inhibitors on interleukin production by human synovial tissue in organ culture: comparison with interleukin-1 synthesis inhibitors J Pharm Pharmacol 1996 48 46 52 8722494 10.1111/j.2042-7158.1996.tb05875.x

[B8] He W Pelleier JP Martel-Pelletier J The synthesis of IL-1β, TNFα and interstitual collagenase (MMP-1) is eicosanoid dependent in human OA synovial membrane explants: interactions with anti-inflammatory cytokines J Rheum 2002 29 546 553 11908571

[B9] Bertolini A Dual acting anti-inflammatory drugs: A reappraisal Pharmacol Res 2001 44 437 450 10.1006/phrs.2001.0872 11735348

[B10] Van Loon IM The golden root: clinical applications of *Scutellaria baicalensi *GEORGI flavanoids as modulators of the inflammatory response Altern Med Rev 1997 2 472 480

[B11] Nakajima T Imanishi M Yamamoto K Cyong JC Hirai K Inhibitory Effect of baicalein, a flavanoid in Scutellaria Root, on eostaxin production by human dermal fibroblasts Planta Med 2001 67 132 135 10.1055/s-2001-11532 11301858

[B12] Zhao S Evaluation of the functional status aspects of health related quality of life of patients with osteoarthritis treated with celecoxib Pharmacotherapy 1999 19 1269 1278 10.1592/phco.19.16.1269.30879 10555933

[B13] Thompson D Pepys MB Wood SP The physiological structure of human C-reactive protein and its complex with phosphocholine Structure 1999 7 2 169 177 10.1016/S0969-2126(99)80023-9 10368284

[B14] Pepys MB Hirschfield GM C-reactive protein: a critical update J Clin Invest 2003 111 12 1805 1812 12813013 10.1172/JCI18921 PMC161431

[B15] Fernandes JC Martel-Pelletier J Pelletier JP The role of cytokines in osteoarthritis pathophysiology Biorheology 2002 39 237 246 12082286

[B16] Lau DC Dhillon B Yan H Szmitko PE Verma S Adipokines: molecular links between obesity and atheroslcerosis Am J Physiol Heart Circ Physiol 2005 288 5 H2031 H2041 10.1152/ajpheart.01058.2004 15653761

[B17] Laporte JR Upper gastrointestinal bleeding in relation to previous use of analgesics and non-steroidal anti- inflammatory drugs Lancet 1991 337 85 89 10.1016/0140-6736(91)90744-A 1670734

[B18] Ong CK Lirk P Tan CH Seymour RA An evidence based update on nonsteroidal anti-inflammatory drugs Clin Med Res 2007 5 19 34 10.3121/cmr.2007.698 17456832 PMC1855338

[B19] Angst F Ann Rheum Dis 2001 60 834 840 11502609 PMC1753825

[B20] Terwee CB J Clin Epidem 2006 59 724 731 10.1016/j.jclinepi.2005.11.019 16765276

[B21] Rockey DC Occult Gastrointestinal Bleeding Gastroenterol Clin North Am 2005 34 4 699 718 21 10.1016/j.gtc.2005.08.010 16303578

[B22] Beg M Occult Gastrointestinal Bleeding: Detection, Interpretation, and Evaluation JIACM 2002 3 2 153 158

